# Supplementary material for: A tissue-specific, Gata6-driven transcriptional program instructs remodeling of the mature arterial tree
Source: eLife. 2017 Sep 27;6:e31362. doi: 10.7554/eLife.31362 (PMC5630260; doi:10.7554/eLife.31362)
Supplement: Supplementary file 5. [file elife-31362-supp5.pdf]

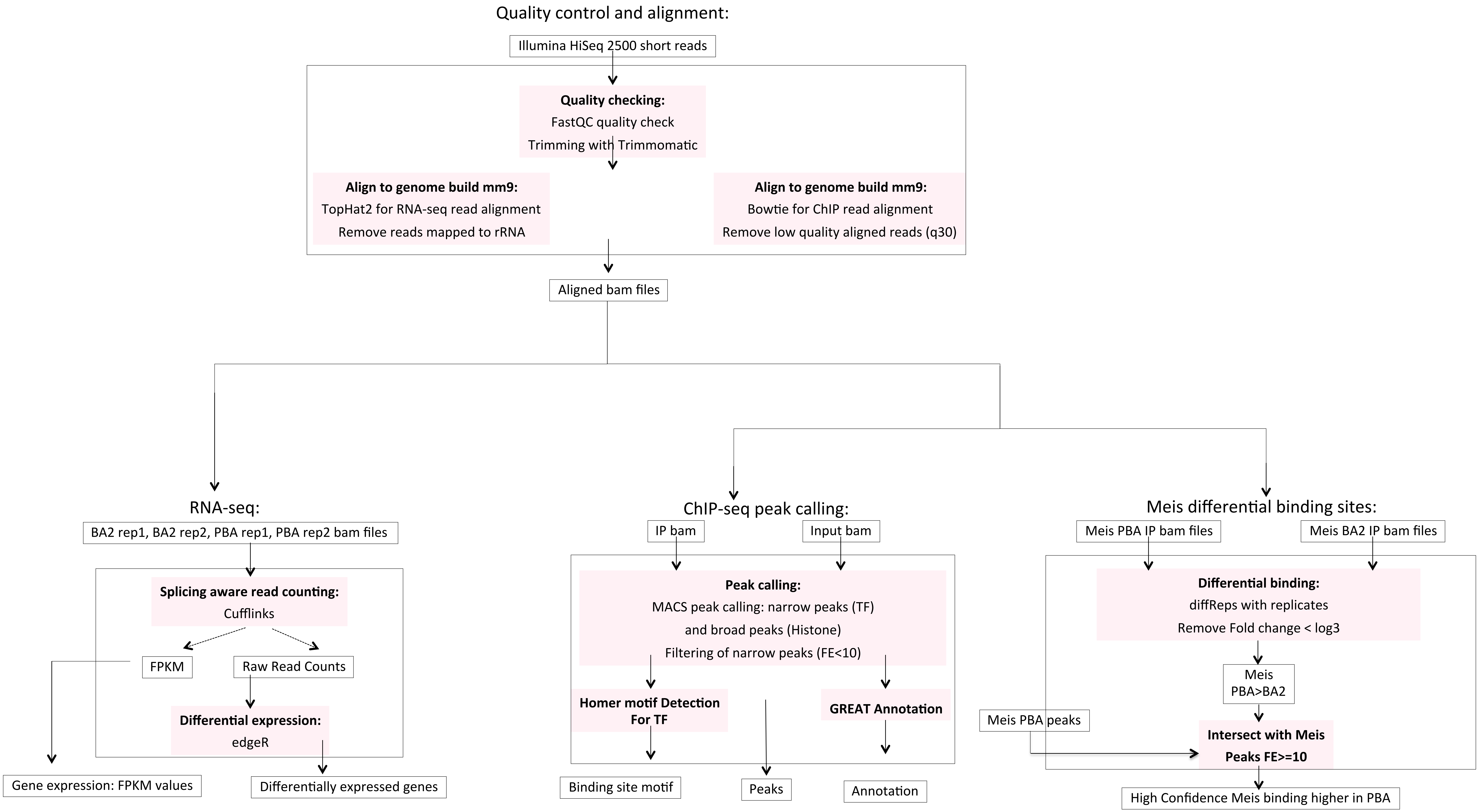
Quality control and alignment:
Illumina HiSeq 2500 short reads
Quality checking:
FastQC quality check
Trimming with Trimmomatic
Align to genome build mm9:
TopHat2 for RNA-seq read alignment
Remove reads mapped to rRNA
Align to genome build mm9:
Bowtie for ChIP read alignment
Remove low quality aligned reads (q30)
Aligned bam files
RNA-seq:
BA2 rep1, BA2 rep2, PBA rep1, PBA rep2 bam files
Splicing aware read counting:
Cufflinks
FPKM
Raw Read Counts
Differential expression:
edgeR
Gene expression: FPKM values
Differentially expressed genes
ChIP-seq peak calling:
IP bam
Input bam
Peak calling:
MACS peak calling: narrow peaks (TF)
and broad peaks (Histone)
Filtering of narrow peaks (FE<10)
Homer motif Detection For TF
GREAT Annotation
Binding site motif
Peaks
Annotation
Meis differential binding sites:
Meis PBA IP bam files
Meis BA2 IP bam files
Differential binding:
diffReps with replicates
Remove Fold change < log3
Meis PBA>BA2
Meis PBA peaks
Intersect with Meis Peaks FE>=10
High Confidence Meis binding higher in PBA
