# Supplementary material for: A tissue-specific, Gata6-driven transcriptional program instructs remodeling of the mature arterial tree
Source: eLife. 2017 Sep 27;6:e31362. doi: 10.7554/eLife.31362 (PMC5630260; doi:10.7554/eLife.31362)
Supplement: Transparent reporting form [file elife-31362-transrepform.docx]

***eLife’s* transparent reporting form**

We encourage authors to provide detailed information *within their submission* to facilitate the interpretation and replication of experiments. If you have any questions, please contact us: [editorial@elifesciences.org](mailto:editorial@elifesciences.org).

**Sample-size estimation**

- You should state whether an appropriate sample size was computed when the study was being designed
- You should state the statistical method of sample size computation and any required assumptions
- If no explicit power analysis was used, you should describe how you decided what sample (replicate) size (number) to use

Please outline where this information can be found within the submission (e.g., page numbers or figure legends), or explain why this information doesn’t apply to your submission:

**Replicates**

- You should report how often each experiment was performed
- You should include a definition of biological versus technical replication
- The data obtained should be provided and sufficient information should be provided to indicate the number of independent biological and/or technical replicates
- If you encountered any outliers, you should describe how these were handled
- Criteria for exclusion/inclusion of data should be clearly stated
- High-throughput sequence data should be uploaded before submission, with a private link for reviewers provided (these are available from both GEO and ArrayExpress)

Please outline where this information can be found within the submission (e.g., page numbers or figure legends), or explain why this information doesn’t apply to your submission:

Each ChIP-Seq and RNA-Seq was performed in duplicates, i.e. two biological replicates for each sample:

ChIP-Seq replicate information reported on Page 19, Line 509-513

RNA-Seq replicate information reported on Page 20, Line 540-541

High-throughput sequence data has been submitted to ArrayExpress

Accession Numbers reported on Page 21, line 572-574

**Statistical reporting**

- Statistical analysis methods should be described and justified
- Raw data should be presented in figures whenever informative to do so (typically when N per group is less than 10)
- For each experiment, you should identify the statistical tests used, exact values of N, definitions of center, methods of multiple test correction, and dispersion and precision measures (e.g., mean, median, SD, SEM, confidence intervals; and, for the major substantive results, a measure of effect size (e.g., Pearson's r, Cohen's d)
- Report exact p-values wherever possible alongside the summary statistics and 95% confidence intervals. These should be reported for all key questions and not only when the p-value is less than 0.05.

Statistical tests for identifying differentially expressed (DE) genes were performed using R package edgeR and multiple test correction was carried out using the R package q-value.

Details mentioned in page 20, lines 549-552.

Fisher Exact test was performed to test the changes in activity of the wildtype and mutant enhancers (line 489). Numbers and corresponding p-values are provided in Figure4-supplement 2 and lines 926-927.

Statistical test for the qPCR data was performed using Zscore transformations.

Details mentioned on page 18, lines 472-477

Please outline where this information can be found within the submission (e.g., page numbers or figure legends), or explain why this information doesn’t apply to your submission:

(For large datasets, or papers with a very large number of statistical tests, you may upload a single table file with tests, Ns, etc., with reference to page numbers in the manuscript.)

**Additional data files (“source data”)**

- We encourage you to upload relevant additional data files, such as numerical data that are represented as a graph in a figure, or as a summary table
- Where provided, these should be in the most useful format, and they can be uploaded as “Source data” files linked to a main figure or table
- Include model definition files including the full list of parameters used
- Include code used for data analysis (e.g., R, MatLab)
- Avoid stating that data files are “available upon request”

Please indicate the figures or tables for which source data files have been provided:

Volcano Plot, bar plot and heatmap in Fig 1C,D and E are based on the numerical data provided in Source Table 1.

Venn Diagram in Fig3B is based on the data provided in Source Table 2

Heatmaps Plotted in Fig 4F,G are based on Source Table 3
